# Supplementary material for: Cell type-specific differences in redox regulation and proliferation after low UVA doses
Source: PLoS One. 2019 Jan 25;14(1):e0205215. doi: 10.1371/journal.pone.0205215 (PMC6347369; doi:10.1371/journal.pone.0205215)
Supplement: S1 Table — (DOCX) [file pone.0205215.s001.docx]

| **Table S1**  **Expression of genes engaged in redox processes in Me45 and HCT116 cells** | | | |
| --- | --- | --- | --- |
| **Gene ID (Entrez Gene)** | **Gene name** | **Expression level in Me45*** | **Expression level in HCT116*** |
| 26574 | AATF | 531 | 553 |
| 1244 | ABCC2 | 28 | 48 |
| 25 | ABL1 | 288 | 182 |
| 59272 | ACE2 | 14 | 13 |
| 8309 | ACOX2 | 39 | 87 |
| 54 | ACP5 | 151 | 36 |
| 92 | ACVR2A | 56 | 121 |
| 100 | ADA | 131 | 91 |
| 8754 | ADAM9 | 102 | 176 |
| 575 | ADGRB1 | 43 | 45 |
| 9370 | ADIPOQ | 18 | 19 |
| 23394 | ADNP | 157 | 230 |
| 22850 | ADNP2 | 80 | 156 |
| 183 | AGT | 37 | 30 |
| 185 | AGTR1 | 22 | 20 |
| 186 | AGTR2 | 17 | 15 |
| 199 | AIF1 | 92 | 60 |
| 9131 | AIFM1 | 305 | 270 |
| 8644 | AKR1C3 | 12 | 10 |
| 207 | AKT1 | 236 | 121 |
| 210 | ALAD | 62 | 61 |
| 213 | ALB | 11 | 10 |
| 221 | ALDH3B1 | 52 | 43 |
| 8846 | ALKBH1 | 136 | 441 |
| 239 | ALOX12 | 25 | 20 |
| 10218 | ANGPTL7 | 419 | 11 |
| 26287 | ANKRD2 | 27 | 33 |
| 55131 | ANKZF1 | 77 | 88 |
| 301 | ANXA1 | 222 | 1032 |
| 328 | APEX1 | 570 | 1545 |
| 337 | APOA4 | 38 | 44 |
| 347 | APOD | 3027 | 34 |
| 348 | APOE | 243 | 85 |
| 55937 | APOM | 52 | 38 |
| 351 | APP | 203 | 125 |
| 54840 | APTX | 108 | 184 |
| 358 | AQP1 | 23 | 22 |
| 374 | AREG | 13 | 175 |
| 378 | ARF4 | 622 | 601 |
| 383 | ARG1 | 18 | 16 |
| 384 | ARG2 | 28 | 39 |
| 10550 | ARL6IP5 | 235 | 212 |
| 405 | ARNT | 29 | 29 |
| 406 | ARNTL | 53 | 98 |
| 445 | ASS1 | 20 | 68 |
| 9474 | ATG5 | 59 | 142 |
| 10533 | ATG7 | 68 | 45 |
| 475 | ATOX1 | 3337 | 296 |
| 23400 | ATP13A2 | 101 | 46 |
| 488 | ATP2A2 | 268 | 146 |
| 493 | ATP2B4 | 69 | 58 |
| 10159 | ATP6AP2 | 404 | 231 |
| 538 | ATP7A | 55 | 25 |
| 93974 | ATPIF1 | 147 | 664 |
| 8455 | ATRN | 60 | 55 |
| 558 | AXL | 36 | 82 |
| 572 | BAD | 64 | 106 |
| 9529 | BAG5 | 45 | 73 |
| 596 | BCL2 | 24 | 25 |
| 613 | BCR | 82 | 40 |
| 8678 | BECN1 | 136 | 148 |
| 637 | BID | 82 | 206 |
| 329 | BIRC2 | 281 | 298 |
| 655 | BMP7 | 98 | 32 |
| 664 | BNIP3 | 188 | 195 |
| 672 | BRCA1 | 54 | 113 |
| 55290 | BRF2 | 84 | 89 |
| 683 | BST1 | 19 | 27 |
| 694 | BTG1 | 522 | 153 |
| 695 | BTK | 40 | 33 |
| 761 | CA3 | 9 | 9 |
| 801 | CALM1 | 135 | 221 |
| 10645 | CAMKK2 | 75 | 49 |
| 836 | CASP3 | 46 | 67 |
| 847 | CAT | 403 | 241 |
| 857 | CAV1 | 109 | 583 |
| 859 | CAV3 | 58 | 56 |
| 57332 | CBX8 | 66 | 60 |
| 6363 | CCL19 | 25 | 23 |
| 6347 | CCL2 | 17 | 17 |
| 6364 | CCL20 | 22 | 19 |
| 890 | CCNA2 | 100 | 203 |
| 891 | CCNB1 | 307 | 955 |
| 1236 | CCR7 | 20 | 17 |
| 9973 | CCS | 115 | 64 |
| 57126 | CD177 | 67 | 43 |
| 947 | CD34 | 44 | 33 |
| 948 | CD36 | 22 | 16 |
| 952 | CD38 | 43 | 37 |
| 972 | CD74 | 3634 | 37 |
| 983 | CDK1 | 179 | 592 |
| 1017 | CDK2 | 565 | 252 |
| 1026 | CDKN1A | 399 | 351 |
| 8837 | CFLAR | 63 | 57 |
| 1137 | CHRNA4 | 27 | 32 |
| 1147 | CHUK | 26 | 94 |
| 1193 | CLIC2 | 15 | 18 |
| 1191 | CLU | 21 | 140 |
| 1268 | CNR1 | 23 | 23 |
| 1269 | CNR2 | 72 | 41 |
| 1277 | COL1A1 | 20 | 21 |
| 1373 | CPS1 | 42 | 42 |
| 1398 | CRK | 104 | 222 |
| 1401 | CRP | 18 | 18 |
| 1410 | CRYAB | 84 | 38 |
| 1421 | CRYGD | 34 | 29 |
| 1471 | CST3 | 375 | 210 |
| 1490 | CTGF | 31 | 40 |
| 1499 | CTNNB1 | 267 | 690 |
| 1497 | CTNS | 91 | 67 |
| 51167 | CYB5R4 | 71 | 106 |
| 1535 | CYBA | 401 | 398 |
| 1536 | CYBB | 21 | 21 |
| 54205 | CYCS | 1960 | 1581 |
| 1543 | CYP1A1 | 47 | 25 |
| 1544 | CYP1A2 | 61 | 41 |
| 1545 | CYP1B1 | 19 | 23 |
| 1571 | CYP2E1 | 22 | 21 |
| 3491 | CYR61 | 36 | 181 |
| 1612 | DAPK1 | 70 | 27 |
| 23576 | DDAH1 | 26 | 280 |
| 23564 | DDAH2 | 110 | 141 |
| 54541 | DDIT4 | 338 | 221 |
| 1718 | DHCR24 | 91 | 234 |
| 10202 | DHRS2 | 27 | 189 |
| 56616 | DIABLO | 376 | 511 |
| 1756 | DMD | 18 | 14 |
| 1759 | DNM1 | 58 | 41 |
| 1785 | DNM2 | 143 | 73 |
| 26052 | DNM3 | 20 | 18 |
| 1800 | DPEP1 | 44 | 39 |
| 1816 | DRD5 | 39 | 23 |
| 53905 | DUOX1 | 23 | 21 |
| 50506 | DUOX2 | 26 | 23 |
| 1843 | DUSP1 | 99 | 101 |
| 8655 | DYNLL1 | 1159 | 1644 |
| 1894 | ECT2 | 39 | 184 |
| 1906 | EDN1 | 39 | 33 |
| 1938 | EEF2 | 1924 | 1295 |
| 1956 | EGFR | 47 | 39 |
| 54583 | EGLN1 | 58 | 55 |
| 2019 | EN1 | 27 | 10 |
| 2021 | ENDOG | 122 | 111 |
| 2034 | EPAS1 | 55 | 53 |
| 57669 | EPB41L5 | 29 | 26 |
| 2052 | EPHX1 | 86 | 91 |
| 2053 | EPHX2 | 37 | 58 |
| 8288 | EPX | 41 | 37 |
| 2067 | ERCC1 | 234 | 139 |
| 2068 | ERCC2 | 86 | 72 |
| 2071 | ERCC3 | 112 | 189 |
| 2074 | ERCC6 | 33 | 20 |
| 1161 | ERCC8 | 69 | 97 |
| 30001 | ERO1A | 45 | 57 |
| 2099 | ESR1 | 31 | 29 |
| 2110 | ETFDH | 28 | 40 |
| 2113 | ETS1 | 76 | 32 |
| 2119 | ETV5 | 214 | 117 |
| 2146 | EZH2 | 43 | 235 |
| 2147 | F2 | 27 | 26 |
| 2150 | F2RL1 | 23 | 206 |
| 2168 | FABP1 | 16 | 18 |
| 2176 | FANCC | 60 | 94 |
| 10516 | FBLN5 | 126 | 25 |
| 25793 | FBXO7 | 1807 | 360 |
| 55294 | FBXW7 | 34 | 60 |
| 2208 | FCER2 | 21 | 20 |
| 2241 | FER | 67 | 81 |
| 2253 | FGF8 | 9 | 9 |
| 2281 | FKBP1B | 24 | 59 |
| 2326 | FMO1 | 31 | 26 |
| 2327 | FMO2 | 22 | 19 |
| 2328 | FMO3 | 11 | 11 |
| 2329 | FMO4 | 93 | 62 |
| 2330 | FMO5 | 20 | 18 |
| 388714 | FMO6P | 30 | 28 |
| 2353 | FOS | 167 | 195 |
| 8061 | FOSL1 | 244 | 251 |
| 2305 | FOXM1 | 269 | 166 |
| 2308 | FOXO1 | 38 | 49 |
| 2309 | FOXO3 | 146 | 230 |
| 2357 | FPR1 | 38 | 25 |
| 2358 | FPR2 | 23 | 22 |
| 79068 | FTO | 166 | 161 |
| 2530 | FUT8 | 62 | 55 |
| 2395 | FXN | 35 | 82 |
| 8321 | FZD1 | 45 | 30 |
| 2539 | G6PD | 154 | 104 |
| 1647 | GADD45A | 65 | 309 |
| 8729 | GBF1 | 155 | 66 |
| 2637 | GBX2 | 17 | 20 |
| 2643 | GCH1 | 14 | 261 |
| 2644 | GCHFR | 68 | 114 |
| 2729 | GCLC | 60 | 95 |
| 2730 | GCLM | 50 | 170 |
| 9573 | GDF3 | 49 | 34 |
| 2717 | GLA | 404 | 461 |
| 51022 | GLRX2 | 151 | 330 |
| 27165 | GLS2 | 28 | 28 |
| 2775 | GNAO1 | 31 | 26 |
| 2861 | GPR37 | 22 | 13 |
| 9283 | GPR37L1 | 27 | 28 |
| 2877 | GPX2 | 46 | 38 |
| 2878 | GPX3 | 29 | 25 |
| 2879 | GPX4 | 1089 | 1332 |
| 2880 | GPX5 | 27 | 25 |
| 2882 | GPX7 | 22 | 17 |
| 2885 | GRB2 | 162 | 159 |
| 2902 | GRIN1 | 40 | 36 |
| 2936 | GSR | 133 | 104 |
| 2937 | GSS | 449 | 301 |
| 2938 | GSTA1 | 23 | 19 |
| 373156 | GSTK1 | 734 | 301 |
| 9446 | GSTO1 | 2038 | 1486 |
| 2950 | GSTP1 | 1042 | 580 |
| 2954 | GSTZ1 | 188 | 133 |
| 2982 | GUCY1A3 | 51 | 33 |
| 2983 | GUCY1B3 | 31 | 17 |
| 9555 | H2AFY | 351 | 400 |
| 54363 | HAO1 | 22 | 20 |
| 3043 | HBB | 60 | 35 |
| 3065 | HDAC1 | 90 | 233 |
| 3066 | HDAC2 | 137 | 289 |
| 9759 | HDAC4 | 59 | 24 |
| 10013 | HDAC6 | 61 | 43 |
| 3280 | HES1 | 81 | 152 |
| 3082 | HGF | 16 | 15 |
| 3091 | HIF1A | 694 | 493 |
| 3099 | HK2 | 148 | 50 |
| 3162 | HMOX1 | 101 | 62 |
| 3163 | HMOX2 | 181 | 163 |
| 3184 | HNRNPD | 119 | 576 |
| 3240 | HP | 19 | 27 |
| 3297 | HSF1 | 59 | 55 |
| 3320 | HSP90AA1 | 1349 | 3469 |
| 3326 | HSP90AB1 | 1664 | 3456 |
| 3303 | HSPA1A | 235 | 10 |
| 3304 | HSPA1B | 59 | 483 |
| 10808 | HSPH1 | 108 | 139 |
| 3357 | HTR2B | 13 | 15 |
| 27429 | HTRA2 | 161 | 102 |
| 3373 | HYAL1 | 44 | 23 |
| 8692 | HYAL2 | 221 | 174 |
| 3383 | ICAM1 | 169 | 39 |
| 3417 | IDH1 | 734 | 495 |
| 3458 | IFNG | 16 | 17 |
| 3586 | IL10 | 18 | 16 |
| 8807 | IL18RAP | 25 | 26 |
| 29949 | IL19 | 64 | 32 |
| 3553 | IL1B | 53 | 47 |
| 3569 | IL6 | 24 | 25 |
| 55364 | IMPACT | 23 | 38 |
| 3630 | INS | 12 | 15 |
| 3643 | INSR | 32 | 34 |
| 26034 | IPCEF1 | 28 | 27 |
| 3684 | ITGAM | 46 | 19 |
| 3689 | ITGB2 | 24 | 23 |
| 3717 | JAK2 | 15 | 21 |
| 3725 | JUN | 31 | 40 |
| 3741 | KCNA5 | 28 | 21 |
| 51780 | KDM3B | 140 | 232 |
| 7403 | KDM6A | 16 | 24 |
| 23135 | KDM6B | 76 | 66 |
| 3791 | KDR | 22 | 20 |
| 8570 | KHSRP | 175 | 140 |
| 10365 | KLF2 | 25 | 74 |
| 9314 | KLF4 | 34 | 51 |
| 3840 | KPNA4 | 30 | 42 |
| 3845 | KRAS | 75 | 97 |
| 3848 | KRT1 | 28 | 23 |
| 3952 | LEP | 22 | 24 |
| 3975 | LHX1 | 25 | 28 |
| 11019 | LIAS | 41 | 132 |
| 9361 | LONP1 | 489 | 406 |
| 4025 | LPO | 41 | 32 |
| 4040 | LRP6 | 52 | 52 |
| 4056 | LTC4S | 35 | 30 |
| 10434 | LYPLA1 | 115 | 520 |
| 4129 | MAOB | 60 | 95 |
| 6416 | MAP2K4 | 50 | 132 |
| 5608 | MAP2K6 | 36 | 28 |
| 4217 | MAP3K5 | 23 | 24 |
| 5594 | MAPK1 | 173 | 160 |
| 1432 | MAPK14 | 85 | 123 |
| 5595 | MAPK3 | 170 | 101 |
| 5598 | MAPK7 | 74 | 49 |
| 5599 | MAPK8 | 37 | 42 |
| 5601 | MAPK9 | 106 | 129 |
| 4137 | MAPT | 52 | 69 |
| 4151 | MB | 67 | 57 |
| 4153 | MBL2 | 13 | 10 |
| 4170 | MCL1 | 80 | 143 |
| 79772 | MCTP1 | 23 | 13 |
| 4193 | MDM2 | 26 | 21 |
| 4204 | MECP2 | 70 | 50 |
| 9833 | MELK | 199 | 565 |
| 4233 | MET | 43 | 81 |
| 4255 | MGMT | 89 | 46 |
| 4258 | MGST2 | 170 | 142 |
| 4259 | MGST3 | 1334 | 1093 |
| 4277 | MICB | 53 | 207 |
| 4323 | MMP14 | 387 | 75 |
| 4314 | MMP3 | 25 | 19 |
| 4318 | MMP9 | 53 | 52 |
| 4353 | MPO | 20 | 22 |
| 4358 | MPV17 | 394 | 175 |
| 4436 | MSH2 | 74 | 294 |
| 4482 | MSRA | 83 | 110 |
| 51734 | MSRB1 | 213 | 244 |
| 22921 | MSRB2 | 113 | 77 |
| 4504 | MT3 | 19 | 38 |
| 4520 | MTF1 | 58 | 82 |
| 2475 | MTOR | 143 | 159 |
| 4548 | MTR | 65 | 74 |
| 4602 | MYB | 25 | 87 |
| 4613 | MYCN | 24 | 27 |
| 4656 | MYOG | 52 | 58 |
| 10135 | NAMPT | 561 | 115 |
| 4688 | NCF2 | 20 | 22 |
| 4689 | NCF4 | 23 | 16 |
| 79625 | NDNF | 27 | 16 |
| 51079 | NDUFA13 | 1120 | 745 |
| 4700 | NDUFA6 | 220 | 445 |
| 4710 | NDUFB4 | 1276 | 1843 |
| 4719 | NDUFS1 | 212 | 382 |
| 4720 | NDUFS2 | 866 | 805 |
| 4722 | NDUFS3 | 873 | 765 |
| 4724 | NDUFS4 | 311 | 507 |
| 4728 | NDUFS8 | 203 | 385 |
| 79661 | NEIL1 | 38 | 43 |
| 10276 | NET1 | 31 | 74 |
| 4760 | NEUROD1 | 9 | 12 |
| 4779 | NFE2L1 | 424 | 282 |
| 4780 | NFE2L2 | 215 | 212 |
| 8382 | NME5 | 44 | 41 |
| 51314 | NME8 | 15 | 14 |
| 23520 | NNT | 59 | 11 |
| 10392 | NOD1 | 106 | 79 |
| 64127 | NOD2 | 24 | 21 |
| 8996 | NOL3 | 42 | 74 |
| 4841 | NONO | 610 | 1097 |
| 4842 | NOS1 | 53 | 48 |
| 9722 | NOS1AP | 62 | 58 |
| 4843 | NOS2 | 49 | 75 |
| 4846 | NOS3 | 39 | 46 |
| 51070 | NOSIP | 127 | 300 |
| 27035 | NOX1 | 30 | 26 |
| 50508 | NOX3 | 17 | 16 |
| 50507 | NOX4 | 12 | 11 |
| 79400 | NOX5 | 49 | 56 |
| 4883 | NPR3 | 26 | 22 |
| 4887 | NPY2R | 14 | 16 |
| 1728 | NQO1 | 1093 | 306 |
| 4929 | NR4A2 | 17 | 57 |
| 8013 | NR4A3 | 27 | 23 |
| 4521 | NUDT1 | 275 | 241 |
| 318 | NUDT2 | 53 | 120 |
| 9688 | NUP93 | 203 | 486 |
| 64359 | NXN | 128 | 291 |
| 4968 | OGG1 | 30 | 35 |
| 29948 | OSGIN1 | 72 | 67 |
| 734 | OSGIN2 | 31 | 31 |
| 55074 | OXR1 | 31 | 36 |
| 9943 | OXSR1 | 204 | 186 |
| 5025 | P2RX4 | 158 | 107 |
| 5027 | P2RX7 | 169 | 20 |
| 5034 | P4HB | 1677 | 827 |
| 5071 | PARK2 | 21 | 21 |
| 11315 | PARK7 | 1539 | 1553 |
| 55486 | PARL | 498 | 425 |
| 142 | PARP1 | 1258 | 456 |
| 5074 | PAWR | 40 | 61 |
| 5076 | PAX2 | 24 | 23 |
| 5080 | PAX6 | 38 | 46 |
| 7703 | PCGF2 | 117 | 89 |
| 5111 | PCNA | 243 | 2403 |
| 11235 | PDCD10 | 324 | 565 |
| 201626 | PDE12 | 24 | 22 |
| 5151 | PDE8A | 89 | 179 |
| 5155 | PDGFB | 33 | 34 |
| 80310 | PDGFD | 34 | 19 |
| 5156 | PDGFRA | 23 | 20 |
| 5159 | PDGFRB | 33 | 40 |
| 64714 | PDIA2 | 25 | 27 |
| 5163 | PDK1 | 41 | 29 |
| 5164 | PDK2 | 76 | 73 |
| 5165 | PDK3 | 58 | 34 |
| 5166 | PDK4 | 22 | 16 |
| 9124 | PDLIM1 | 212 | 406 |
| 3651 | PDX1 | 20 | 18 |
| 5179 | PENK | 29 | 24 |
| 5229 | PGGT1B | 23 | 33 |
| 55022 | PID1 | 30 | 15 |
| 65018 | PINK1 | 194 | 79 |
| 5311 | PKD2 | 54 | 43 |
| 22925 | PLA2R1 | 10 | 9 |
| 59338 | PLEKHA1 | 49 | 199 |
| 1263 | PLK3 | 68 | 71 |
| 5366 | PMAIP1 | 17 | 144 |
| 5371 | PML | 63 | 38 |
| 11284 | PNKP | 93 | 71 |
| 5445 | PON2 | 341 | 318 |
| 5446 | PON3 | 40 | 29 |
| 5447 | POR | 82 | 101 |
| 10891 | PPARGC1A | 52 | 16 |
| 10105 | PPIF | 340 | 378 |
| 5516 | PPP2CB | 40 | 51 |
| 5536 | PPP5C | 125 | 181 |
| 5547 | PRCP | 337 | 264 |
| 5052 | PRDX1 | 1696 | 2571 |
| 7001 | PRDX2 | 591 | 1449 |
| 10935 | PRDX3 | 792 | 1468 |
| 10549 | PRDX4 | 942 | 1304 |
| 9588 | PRDX6 | 563 | 1001 |
| 10394 | PRG3 | 38 | 27 |
| 5562 | PRKAA1 | 16 | 39 |
| 5580 | PRKCD | 230 | 37 |
| 5587 | PRKD1 | 108 | 15 |
| 5593 | PRKG2 | 52 | 50 |
| 8575 | PRKRA | 92 | 144 |
| 5621 | PRNP | 148 | 287 |
| 5626 | PRODH | 33 | 37 |
| 79899 | PRR5L | 15 | 17 |
| 5660 | PSAP | 1790 | 328 |
| 5663 | PSEN1 | 53 | 60 |
| 11168 | PSIP1 | 105 | 256 |
| 5693 | PSMB5 | 755 | 1486 |
| 5740 | PTGIS | 23 | 18 |
| 5742 | PTGS1 | 35 | 25 |
| 5743 | PTGS2 | 10 | 13 |
| 2185 | PTK2B | 41 | 40 |
| 5796 | PTPRK | 104 | 356 |
| 5798 | PTPRN | 31 | 34 |
| 5805 | PTS | 193 | 417 |
| 5806 | PTX3 | 15 | 9 |
| 7837 | PXDN | 80 | 28 |
| 5829 | PXN | 145 | 95 |
| 5831 | PYCR1 | 263 | 231 |
| 79912 | PYROXD1 | 65 | 74 |
| 5873 | RAB27A | 203 | 63 |
| 5879 | RAC1 | 1080 | 954 |
| 5880 | RAC2 | 46 | 105 |
| 10399 | RACK1 | 4777 | 5545 |
| 5893 | RAD52 | 20 | 30 |
| 11030 | RBPMS | 53 | 24 |
| 5970 | RELA | 160 | 96 |
| 55312 | RFK | 77 | 149 |
| 9104 | RGN | 26 | 27 |
| 10636 | RGS14 | 50 | 42 |
| 387 | RHOA | 1445 | 2265 |
| 388 | RHOB | 470 | 208 |
| 8737 | RIPK1 | 51 | 85 |
| 10193 | RNF41 | 44 | 64 |
| 6095 | RORA | 24 | 15 |
| 6098 | ROS1 | 19 | 25 |
| 6188 | RPS3 | 5032 | 6182 |
| 51389 | RWDD1 | 260 | 322 |
| 6280 | S100A9 | 34 | 33 |
| 51435 | SCARA3 | 34 | 61 |
| 949 | SCARB1 | 850 | 81 |
| 7356 | SCGB1A1 | 22 | 19 |
| 6331 | SCN5A | 28 | 50 |
| 6382 | SDC1 | 35 | 37 |
| 57001 | SDHAF3 | 21 | 46 |
| 6414 | SELENOP | 15 | 15 |
| 6415 | SELENOW | 585 | 726 |
| 27244 | SESN1 | 126 | 61 |
| 23064 | SETX | 78 | 84 |
| 6421 | SFPQ | 75 | 132 |
| 6441 | SFTPD | 26 | 23 |
| 101010 | SGK2 | 31 | 26 |
| 9644 | SH3PXD2A | 22 | 25 |
| 23729 | SHPK | 43 | 37 |
| 23411 | SIRT1 | 38 | 126 |
| 22933 | SIRT2 | 216 | 228 |
| 23410 | SIRT3 | 100 | 65 |
| 23408 | SIRT5 | 69 | 76 |
| 6571 | SLC18A2 | 17 | 16 |
| 9962 | SLC23A2 | 73 | 31 |
| 29957 | SLC25A24 | 35 | 92 |
| 55532 | SLC30A10 | 23 | 13 |
| 6532 | SLC6A4 | 43 | 30 |
| 23657 | SLC7A11 | 31 | 37 |
| 6542 | SLC7A2 | 17 | 23 |
| 6546 | SLC8A1 | 29 | 28 |
| 4088 | SMAD3 | 55 | 55 |
| 4089 | SMAD4 | 18 | 27 |
| 6622 | SNCA | 159 | 38 |
| 6640 | SNTA1 | 158 | 60 |
| 6647 | SOD1 | 1736 | 2903 |
| 6648 | SOD2 | 60 | 45 |
| 6649 | SOD3 | 54 | 64 |
| 64321 | SOX17 | 17 | 15 |
| 6690 | SPINK1 | 17 | 15 |
| 6697 | SPR | 381 | 289 |
| 6714 | SRC | 43 | 42 |
| 23648 | SSBP3 | 106 | 122 |
| 6770 | STAR | 28 | 29 |
| 6772 | STAT1 | 78 | 159 |
| 6774 | STAT3 | 240 | 87 |
| 6778 | STAT6 | 173 | 77 |
| 6780 | STAU1 | 494 | 380 |
| 8614 | STC2 | 29 | 62 |
| 9263 | STK17A | 36 | 36 |
| 8428 | STK24 | 458 | 375 |
| 10494 | STK25 | 229 | 76 |
| 51765 | STK26 | 44 | 97 |
| 2054 | STX2 | 77 | 110 |
| 6810 | STX4 | 89 | 87 |
| 6821 | SUOX | 73 | 66 |
| 6850 | SYK | 25 | 23 |
| 23334 | SZT2 | 40 | 23 |
| 6869 | TACR1 | 24 | 27 |
| 6898 | TAT | 24 | 28 |
| 6911 | TBX6 | 45 | 41 |
| 7014 | TERF2 | 97 | 232 |
| 7015 | TERT | 35 | 56 |
| 7020 | TFAP2A | 275 | 48 |
| 7040 | TGFB1 | 48 | 40 |
| 7048 | TGFBR2 | 74 | 31 |
| 7057 | THBS1 | 22 | 20 |
| 148022 | TICAM1 | 32 | 37 |
| 57103 | TIGAR | 123 | 85 |
| 57707 | TLDC1 | 21 | 21 |
| 7097 | TLR2 | 24 | 19 |
| 7099 | TLR4 | 26 | 24 |
| 7100 | TLR5 | 29 | 24 |
| 10333 | TLR6 | 25 | 23 |
| 10959 | TMED2 | 310 | 446 |
| 54929 | TMEM161A | 408 | 281 |
| 7124 | TNF | 33 | 25 |
| 7128 | TNFAIP3 | 101 | 43 |
| 1861 | TOR1A | 108 | 179 |
| 7157 | TP53 | 89 | 23 |
| 7168 | TPM1 | 58 | 77 |
| 7173 | TPO | 19 | 25 |
| 6434 | TRA2B | 113 | 382 |
| 7186 | TRAF2 | 75 | 68 |
| 10131 | TRAP1 | 434 | 989 |
| 8989 | TRPA1 | 24 | 20 |
| 7225 | TRPC6 | 15 | 15 |
| 7226 | TRPM2 | 49 | 121 |
| 7442 | TRPV1 | 27 | 28 |
| 7248 | TSC1 | 74 | 105 |
| 706 | TSPO | 757 | 270 |
| 11334 | TUSC2 | 128 | 160 |
| 7291 | TWIST1 | 37 | 10 |
| 7295 | TXN | 949 | 2270 |
| 25828 | TXN2 | 238 | 239 |
| 10628 | TXNIP | 346 | 32 |
| 9352 | TXNL1 | 314 | 1281 |
| 7296 | TXNRD1 | 758 | 693 |
| 10587 | TXNRD2 | 62 | 55 |
| 114112 | TXNRD3 | 70 | 69 |
| 29914 | UBIAD1 | 58 | 65 |
| 7349 | UCN | 30 | 45 |
| 7350 | UCP1 | 23 | 22 |
| 7351 | UCP2 | 51 | 33 |
| 7352 | UCP3 | 29 | 36 |
| 7409 | VAV1 | 36 | 70 |
| 7415 | VCP | 334 | 276 |
| 8876 | VNN1 | 11 | 10 |
| 7444 | VRK2 | 103 | 230 |
| 8976 | WASL | 29 | 37 |
| 7471 | WNT1 | 23 | 17 |
| 51384 | WNT16 | 44 | 170 |
| 7486 | WRN | 26 | 61 |
| 7498 | XDH | 82 | 57 |
| 7507 | XPA | 60 | 79 |
| 7515 | XRCC1 | 80 | 99 |
| 80149 | ZC3H12A | 104 | 57 |
| 7755 | ZNF205 | 25 | 53 |
| 11179 | ZNF277 | 107 | 108 |
| 51157 | ZNF580 | 104 | 66 |
| ^*^arbitrary units reflect normalized data from microarray experiment. | | | |
