# Supplementary material for: Cell type-specific differences in redox regulation and proliferation after low UVA doses
Source: PLoS One. 2019 Jan 25;14(1):e0205215. doi: 10.1371/journal.pone.0205215 (PMC6347369; doi:10.1371/journal.pone.0205215)
Supplement: S2 Table — (DOCX) [file pone.0205215.s002.docx]

| **Gene** | **Forward primer sequence** | **Reverse primer sequence** |
| --- | --- | --- |
| CAT | TAAGACTGACCAGGGCATC | CAAACCTTGGTGAGATCGAA |
| GSTO1 | GGACGCGTCTAGTCCTGAAG | CAGGTGATGGCAGACTCGTAG |
| GSTP1 | ATGCCGCCCTACACCGTG | CCAGGTGACGCAGGATGG |
| PRDX3 | CAGCCGTTGTCAATGGAGAG | CCAAAGGATAGAAGAAAAGCACC |
| GPX4 | GCCTTCCCGTGTAACCAGT | GCGAACTCTTTGATCTCTTCGT |
| RPL41 | TCCTGCGTTGGGATTCCGTG | ACGGTGCAACAAGCTAGCGG |
| TXN1 | GTGAAGCAGATCGAGAGCAAG | CGTGGCTGAGAAGTCAACTACTA |
